# Unraveling the Synergistic Mechanisms of Phosphorus Adsorption and Slow-Release on Low-Mg-Loaded Biochar Enabled by KOH Activation

**DOI:** 10.3390/ma18225214

**Published:** 2025-11-18

**Authors:** Fengyun Bu, Lei Han, Hongxia Guo, Yu Liang, Haihong Yan

**Affiliations:** 1 State Key Laboratory of Environmental Criteria and Risk Assessment, Chinese Research Academy of Environmental Sciences, Beijing 100012, China; 2 Research Center of Environmental Pollution Control Engineering Technology, Chinese Research Academy of Environmental Sciences, Beijing 100012, China; 3 College of Materials Science and Engineering, Beijing University of Technology, Beijing 100124, China; 4 Xinkai Environment Investment Co., Ltd., Beijing 101101, China; hanlei726@126.com

**Keywords:** phosphorus recovery, biochar, synergistic modification, adsorption mechanism, fertilizer, agricultural waste

## Abstract

**Highlights:**

**What are the main findings?**

- A novel functional straw biochar (F-SBC) was engineered.
- F-SBC shows superior pH adaptability and regeneration stability.
- Synergistic KOH-activation and Mg-modification strategy was used.

**What are the implications of the main findings?**

- The adsorbent has great potential for practical wastewater treatment.
- Synergistic removal mechanisms including precipitation were revealed.
- Provides a effective way to design high-performance biochar materials.

**Abstract:**

Phosphorus (P) scarcity and pollution demand sustainable recovery strategies. This study engineered a functional straw biochar (F-SBC) from corn straw through synergistic KOH activation and MgCl_2_ modification for efficient P recovery and slow release. Characterization revealed that KOH pretreatment expanded pore size and enhanced MgO loading. Batch adsorption experiments demonstrated F-SBC achieved a remarkable P adsorption capacity of 24.70 ± 0.57 mg·g^−1^, and exhibited > 95% removal efficiency across pH 5~9. Adsorption kinetics followed the pseudo-second-order model, and isotherms fitted the Langmuir model, indicating chemisorption-dominated monolayer adsorption. Mechanistic studies identified synergistic contributions from chemical precipitation, inner-sphere complexation, bi-metallic electrostatic attraction, and physical confinement. F-SBC exhibited slow-release properties, alongside sustained adsorption capacity. Competitive anions (HCO_3_^−^/CO_3_^2−^) significantly promoted desorption, while Cl^−^ showed minimal impact. This KOH/MgCl_2_ co-modification strategy creates a cost-effective, regenerable biochar with superior P recovery and controlled-release potential, advancing sustainable P management from agricultural waste towards a circular bioeconomy.

## 1. Introduction

Phosphorus (P) is an essential element for global food security and ecosystem function, playing a critical role in biological molecules such as nucleic acids, ATP, and phospholipids [1]. Nevertheless, the finite nature of global phosphate rock reserves presents a serious resource security challenge. More than 85% of mined phosphate is used in fertilizer production, and current projections suggest reserves could be depleted within a century under increasing agricultural demand [2,3]. At the same time, inefficient fertilizer application leads to significant phosphorus losses (20~25%) into aquatic environments, contributing to eutrophication and biodiversity decline [4,5]. This dual crisis of resource depletion and environmental pollution underscores the urgent need for sustainable P recovery and recycling technologies.

Agricultural residues, such as corn straw, represent a widely available yet underutilized resource that could help address this challenge. China alone produces over 200 million tons of corn straw annually, with approximately 20% disposed of via open burning, exacerbating air pollution and greenhouse gas emissions [6,7]. Converting this biomass into biochar offers a promising pathway for carbon sequestration and P recovery, given its potential as a porous adsorbent [8]. However, raw biochar exhibits a relatively low capacity for P adsorption (0.59~1.29 mg·g^−1^) and thus is often chemically modified to improve its performance [4].

Among various modification approaches, Mg-impregnated biochar has attracted considerable interest due to the strong interaction between Mg oxides and phosphate anions, as well as its potential use as a slow-release P fertilizer [9]. Despite promising laboratory results, the widespread application of Mg-modified biochar is often constrained by high Mg salt requirements (typically 2.0~5.0 M MgCl_2_), which raise production costs and undermine economic feasibility [10,11,12]. Although alkaline pre-activation is known to enhance biochar porosity, its synergistic effects with subsequent Mg modification have not been systematically quantified [10,13]. Moreover, while previous studies attribute P uptake mainly to precipitation and electrostatic attraction, the molecular scale mechanisms involving KOH-tuned pore structures, MgO dispersion, and the interplay among inner-sphere complexation, electrostatic interactions, and physical confinement remain poorly elucidated [11,12]. This knowledge gap hinders the rational design of biochars with optimized P recovery and controlled-release properties.

Furthermore, many reported biochar adsorbents suffer from limited pH adaptability and interference from common co-existing anions [14]. Crucially, the desorption behavior and the competitive influence of anions such as HCO_3_^−^/CO_3_^2−^ on P release are rarely systematically investigated, which is crucial for predicting the performance of biochar as a fertilizer in real soil environments.

To address these limitations, this study proposes a synergistic KOH-MgCl_2_ modification strategy to engineer a functional biochar (F-SBC) from corn straw for P recovery. We hypothesize that KOH pre-activation will create a porous framework that facilitates highly dispersed MgO loading at a reduced MgCl_2_ dosage, thereby enhancing P adsorption through multiple synergistic mechanisms and enabling controllable release. The specific objectives are to perform the following: (1) characterize the physicochemical properties of raw and modified biochars, focusing on the synergistic effects of KOH and MgCl_2_ treatment; (2) evaluate the P adsorption performance under various conditions, and assess the slow-release behavior and regeneration stability of F-SBC; and (3) elucidate the atomic-level adsorption and desorption mechanisms through a combination of spectroscopic and microscopic techniques.

This work bridges fundamental material engineering with practical environmental application. It not only provides a cost-effective strategy for converting agricultural waste into a high-value adsorbent but also offers deep mechanistic insights that advance the scientific foundation for designing next-generation biochars for sustainable phosphorus management in a circular bioeconomy.

## 2. Materials and Methods

### 2.1. Materials and Preparation of Pristine and Modified Biochars

The corn straw-derived biochar (ash content: 8.43%; pH: 9.46) was obtained from Henan Lizhe Environmental Co., Ltd. (Shangqiu, China). The biochar was washed to reduce ash content, uniformly ground, dehydrated at 105 °C for 24 h, then oven-dried at 80 °C to constant weight, sieved by a 100-mesh sieve to ensure homogeneity.

The sieved samples were loaded into a quartz boat and thermally disintegrated through tubular furnace (Aikexun TF1200-60, Tianjin, China) under oxygen-limited conditions to obtain the pristine biochar (denoted as SBC). The pyrolysis was conducted at 600 °C with a 5 °C·min^−1^ heating rate and a 2 h holding time. SBC was mixed with 1 M KOH at a solid-to-liquid ratio of 1:25, agitated under 25 °C over 6 h, washed to neutrality, and centrifuged, named as K-SBC. SBC was similarly treated with 1.5 M MgCl_2_·6H_2_O (solid-to-liquid ratio: 1:25), followed by washing, centrifugation, and drying, named as M-SBC. Both K-SBC and M-SBC were then subjected to secondary pyrolysis at 600 °C for 2 h under inert conditions to enhance stability. Finally, the composite-modified biochar (F-SBC) was synthesized by impregnating K-SBC with MgCl_2_·6H_2_O solution at a solid-to-liquid ratio of 1:25, repeating the impregnation-pyrolysis cycle.

### 2.2. Adsorption Experiment

P adsorption performance was evaluated through batch experiments using SBC, K-SBC, M-SBC, and F-SBC. The 0.1 g biochar samples were mixed with 50 mL phosphorus solutions in 250 mL flasks and shaken at 180 rpm. The system was maintained at 25 °C to reach adsorption equilibrium. To investigate adsorption mechanisms, three independent variables were conducted as follows: (i) contact time (1~2880 min), (ii) initial phosphorus concentration (1~80 mg·L^−1^), and (iii) solution pH (3, 5, 7, 9, 11). All trials were performed in triplicate. Post-adsorption, supernatants were filtered via 0.45 μm membranes, the residual P concentrations were measured using UV-vis spectrophotometry at 700 nm (Thermo Fisher Scientific L5, Waltham, MA, USA). The adsorption amount (q_e_) and the removel rate (ƞ) were calculated using the following equations:

(1) $$q_{e}\;=\;\frac{(C_{0}\;-\;C_{e})V}{m}$$

(2) $$\eta \;=\frac{C_{0}-C}{C_{0}}\;\times \;100\%$$

where q_e_ is the equilibrium adsorption amount, mg·g^−1^; C_0_ is the initial P mass concentration in the solution before adsorption, mg·L^−1^; C_e_ is the residual P mass concentration in the solution after adsorption, mg·L^−1^; V is the volume of the solution, L; and m is the dosage of the adsorbent, g.

### 2.3. Adsorption Kinetics Experiment

The adsorption kinetics of P were modeled using pseudo-first-order and pseudo-second-order equations. The intraparticle diffusion model was further applied to identify the rate-limiting steps. The applicability of each model was assessed based on the correlation coefficient (R^2^). The models were given by the following:

(3) $$\ln(q_{e}-q_{t})=\ln q_{e\;}-{\;k}_{1}t$$

(4) $$\frac{t}{q_{t}}=\frac{1}{k_{2}q_{e}^{2}}+\frac{t}{q_{e}}$$

(5) $$q_{t}=k_{\mathrm{id}}\;t^{1/2}+C$$

where q_t_ is the adsorption capacity at time t, mg·g^−1^; t is the adsorption time, min; k_1_ and k_2_ are the rate constants of the pseudo-first-order and pseudo-second-order models, g·(mg·min)^−1^; and $k_{\mathrm{id}}$ is the intraparticle diffusion rate constant, mg·g^−1^·min^−^^0.5^.

### 2.4. Adsorption Isotherm Experiments

The Langmuir and Freundlich models were fitted to the adsorption data to obtain the maximum adsorption capacity (q_m_) of the biochars.

The Langmuir and Freundlich model was given by the following:

(6) $$q_{e}\;=\;\frac{q_{m}K_{L}C_{e}}{1\;+{\;K}_{L}C_{e}}$$

(7) $$q_{e}={\;K}_{F}C_{e}^{\frac{1}{n}}$$

where q_m_ is the maximum adsorption capacity (mg·g^−1^), C_e_ is the residual phosphorus concentration (mg·L^−1^), K_L_ is the Langmuir adsorption constant (L·mg^−1^), K_F_ is the Freundlich isotherm constant ((mg·g^−1^)·(mg·L^−1^)^−n^), and n is the adsorption constant. The applicability of each model was assessed using the correlation coefficient, which provides a measure of how well the experimental data fit the model.

### 2.5. Desorption Experiments

P desorption experiments were performed by adding 0.1 g of F-SBC-P and 50 mL of ultrapure water in conical flasks and stirring (180 rpm) for 1440 min under controlled ambient conditions (25 °C). To investigate specific anion effects, comparative experiments were conducted in 1 M solutions of OH^−^, Cl^−^, CO_3_^2−^, and HCO_3_^−^, prepared from their corresponding sodium salts of Analytical Reagent (AR) grade. Ultrapure water was used as the control. Following each experiment cycle, the suspensions were filtered through a membrane for subsequent P measurement. For the study of sequential release characterization, post-filtration residues were air-dried and reintroduced to fresh ultrapure water under identical experimental parameters for seven cycles. Each experiment was repeated three times. The phosphorus release amount (Q) and the release rate (ɛ) were calculated using the following equations:

(8) $$Q\;\;=\;\frac{(\rho_{0}\;-\;\rho_{t})V}{m}$$

(9) $$\varepsilon =\frac{Q-{\;Q}_{0}}{q_{e}}\;\times \;100\%$$

where ρ_0_ and ρ_t_ are the initial and the phosphorus mass concentration in the solution at time t, respectively, mg·L^−1^; V is the volume of the ultrapure water, L; and Q_0_ is the initial phosphorus mass concentration in the solution before release, mg·L^−1^.

### 2.6. Regeneration Experiment

The reusability of F-SBC was evaluated through five consecutive adsorption–desorption cycles. In each cycle, 0.1 g of F-SBC was added to 50 mL of phosphate-containing simulated wastewater (initial concentration: 50 mg·L^−1^) under continuous agitation at 180 rpm until reaching adsorption equilibrium. After adsorption, the adsorbent was recovered by filtration through 0.45 μm membranes, followed by desorption in 50 mL of ultrapure water under identical agitation parameters. The desorbed phosphate concentration in the supernatant was quantified using the above method. The adsorption capacity was determined by Equation (2), desorption capacity by Equation (9).

### 2.7. Characterization Methods

The pH value was determined using the electrode method (HJ 1147-2020). The sample was poured down the beaker wall, and the electrode was immediately immersed. The reading was recorded once stable. The point of zero charge (PZC) was measured by the zeta potential analyzer (Zata, Nano ZS90, Malvern Panalytical, Westborough, MA, USA). The textural properties of the biochar, including its specific surface area, pore volume, and pore size distribution, were characterized via nitrogen physisorption by a Micromeritics ASAP 2420 instrument (Shanghai, China). BET denotes the specific surface area of a solid material, measured in square meters per gram (m^2^·g^−1^). The surface morphology of the biochar before and after modification and adsorption was observed using the scanning electron microscope (SEM, Zeiss sigma360, Oberkochen, Germany), and the mass fractions of C, O, Mg, P, K, were determined using energy-dispersive spectroscopy (EDS, Zeiss sigma360, Oberkochen, Germany). The Fourier transform infrared spectrometer (FT-IR, Thermo Fisher Nicolet iS50, Waltham, MA, USA) was used to analyze the valence bond structure and the changes in surface functional groups of the biochar before and after adsorption. The X-ray diffractometer (XRD, Shimadzu XRD-6100, Kyoto, Japan) was used to analyze the phase structure and infer the crystal phase changes. The analysis of elemental valence states was characterized by X-ray photoelectron spectrometer (XPS, Thermo Scientific EscaLab 250Xi, Waltham, MA, USA) and Raman spectra (Raman, Thermo Fischer DXR, Waltham, MA, USA). The graphitization degree was determined by the ratio of ID/IG (ID: the value of the disorder–band–peak, IG: the value of the graphitic–band–peak). The ID/IG < 1 indicates a high degree of graphitization, while the degree of defect is controlled by a larger ratio.

## 3. Results

### 3.1. Characteristics of Adsorbents

#### 3.1.1. Morphological and Elemental Characterization

**Figure 1:**
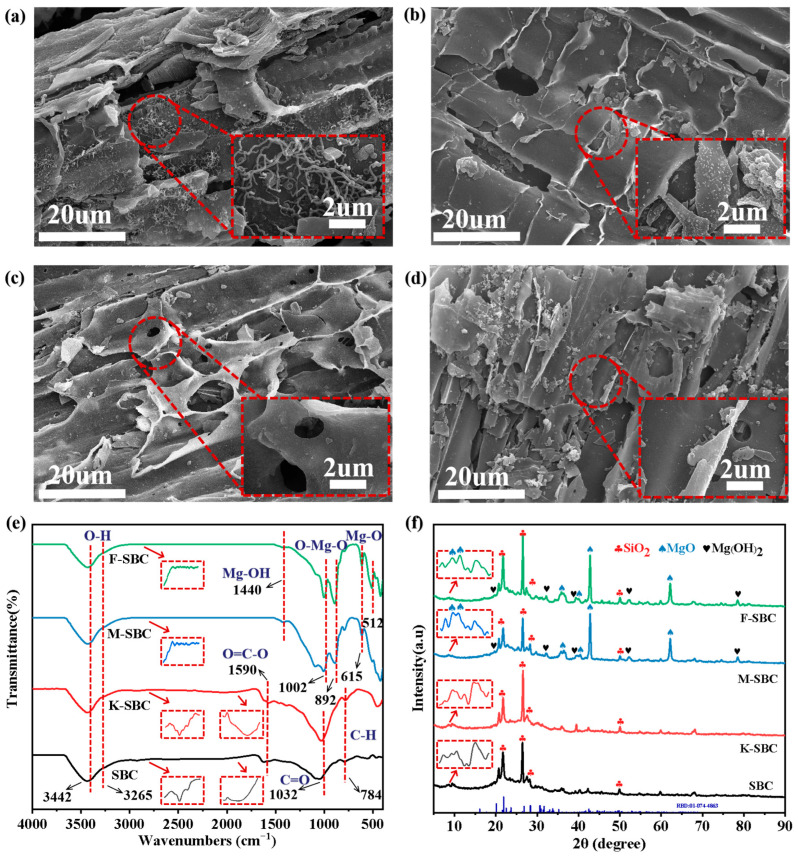
Microstructural and physicochemical characterization of raw and modified biochars. (**a**–**d**) SEM images with magnification of 20 μm and 2 μm, (**a**) SBC, (**b**) K-SBC, (**c**) M-SBC, and (**d**) F-SBC; (**e**) FT-IR spectra at wavelengths from 500 to 4000 cm^−1^ with the scan resolution to 4 cm^−1^; (**f**) XRD patterns with the scan speed to 5°/min, scan angle to 5–90°.

SEM images (Figure 1a–d) were employed to characterize the microstructural morphology of the biochars. The overall porous sheet structure with tubular or lamellar frameworks was maintained across all samples. However, significant differences in surface features were observed. SBC and K-SBC showed relatively smooth surfaces, whereas M-SBC and F-SBC surfaces were coarsened and covered with irregular particulate aggregates. The disappearance of the filamentous structures in modified biochars further indicates surface modification [15]. EDS analysis (Table 1) confirmed elemental shifts. While C, O, Si, and Ca existed in all samples, with contents ranging from 81.1 to 87.4%, 9.8–12.4%, 1.0–2.6%, and 0.1–0.8%, respectively. K-SBC, M-SBC, and F-SBC showed K and Mg signatures. Mg contents reached 6.10% (M-SBC) and 6.90% (F-SBC), verifying successful MgO loading. Higher Mg loading capacity in F-SBC underscores KOH’s role in suppressing particle aggregation [16].

#### 3.1.2. Porous Structure Analysis

Figure S1 presents the nitrogen adsorption–desorption isotherms and the corresponding BJH pore size distribution profiles for the different biochars. The BJH curves revealed pore distributions in the micropore (<2 nm) and mesopore (2~5 nm) ranges for all biochars. Notably, F-SBC showed a pronounced peak at 2~5 nm, indicating a dominance of mesopores compared to the others. Quantitative analysis (Table 1) showed K-SBC achieved the highest BET value (197.03 m^2^·g^−1^) and pore volume (0.1118 cm^3^·g^−1^) due to KOH-induced matrix etching [17]. The BET of M-SBC and F-SBC decreased to 164.54 and 137.62 m^2^·g^−1^, respectively, as a result of MgO deposition. However, F-SBC exhibited a 32.10% larger average pore diameter and 13.04% higher pore volume than SBC, confirming KOH pretreatment expands pore dimensions prior to MgCl_2_ modification. This enhanced mesoporosity facilitates phosphate adsorption (ionic radius ≈ 0.238 nm [18]) via pore filling and surface complexation. The balance between specific surface area and pore accessibility underscores the importance of tailoring the pore architecture for specific adsorbates, with factors beyond porosity also playing a significant role in the overall adsorption capacity [19,20].

#### 3.1.3. Surface Functional Groups

FT-IR spectra in Figure 1e reveal the evolution of characteristic functional groups for SBC and its modified samples. The absorption bands at 3442 cm^−1^ and 3265 cm^−1^ in all samples were attributed to the stretching vibration of hydroxyl groups and bending vibration of interlayer water molecules. The oxygen-containing groups of SBC and K-SBC include the O=C–O (ester group), C=O (carbonyl group), and C–H, which peak at 1590 cm^−1^, 1032 cm^−1^, and 784 cm^−1^ [21]. The intensity of the bands of the oxygen-containing functional groups between SBC and K-SBC suggests that KOH activation preserved surface functional groups and increased the amount of oxygen-containing functional groups. The ester functional group (observed at 1819 cm^−1^) was transformed into an aldehyde group (at 1695 cm^−1^), suggesting the cleavage of ester linkages following KOH treatment. While its impact on the aromatic C–H stretches (2850–3100 cm^−1^) was minimal, these aromatic hydrocarbons vanished after the subsequent Mg modification. For M-SBC and F-SBC, peaks at lower wavenumbers from 400 to 800 cm^−1^ and 1440 cm^−1^ corresponded to bending vibration of Mg–O, Mg–O–Mg, and Mg–OH [22]. M-SBC and F-SBC exhibited sharp peaks at 615 cm^−1^, 892 cm^−1^, and 1002 cm^−1^, suggesting MgO is the primary structure onto modified biochars by magnesium [23]. F-SBC showed intensified peaks in the 500–650 cm^−1^ range, signifying higher MgO loading compared to M-SBC. These observations agree with EDS data, confirming that MgCl_2_ modification replaced organic groups with MgO/Mg(OH)_2_ structures [24,25].

#### 3.1.4. Crystalline Phase Composition

The XRD patterns in Figure 1f revealed distinct structural features of the biochars, combining crystalline and amorphous phases. All samples exhibited characteristic diffraction peaks at 20.4°, 26.6°, and 50.2°, consistent with SiO_2_ crystals and graphitizing carbon [26]. Notably, M-SBC and F-SBC displayed additional structural change: (1) A broad amorphous hump at 15–30° indicated disordered carbon matrices, which could be attributed to the dispersion of Mg-based components; (2) Sharp peaks at 10.1, 10.3°, 36.7°, 39.1°, 43.3°, and 62.5° were indexed to crystalline MgO [27]. The narrow peak widths demonstrated high crystallinity and phase purity of MgO. New peaks at 21.8°, 52.3°, and 78.8° corresponded to Mg(OH)_2_, suggesting partial hydration of MgO during modification. Stronger Mg(OH)_2_ signals in F-SBC reflected KOH-enhanced surface reactivity during hydration. This hydrated phase improves ion exchange efficiency, synergizing with dispersed MgO crystallites to enhance functionality [25].

### 3.2. Adsorption Performance of Different Biochars

#### 3.2.1. Adsorption Kinetics

**Figure 2:**
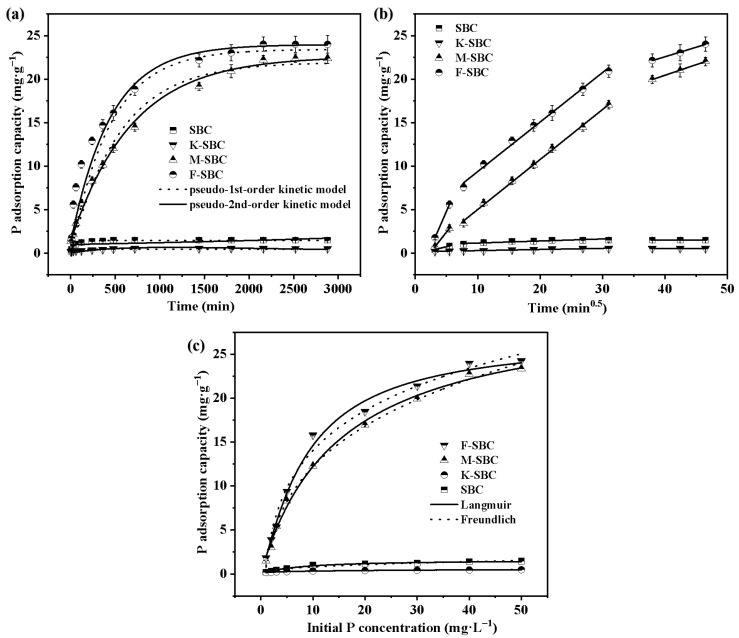
Pseudo-first-order, pseudo-second-order kinetic curves of pristine material and the modified materials (**a**); intra-particle diffusion kinetic curves of pristine material and the modified materials (**b**); Langmuir, Freundlich isotherms curves of pristine material and the modified materials (**c**).

The adsorption kinetics of P on various biochars are presented in Figure 2a. At equilibrium, the maximum adsorption capacities of SBC, K-SBC, M-SBC, and F-SBC were 1.43 ± 0.02 mg·g^−1^, 0.57 ± 0.02 mg·g^−1^, 21.46 ± 0.53 mg·g^−1^, and 24.70 ± 0.57 mg·g^−1^, corresponding to P removal efficiencies of 4.25 ± 0.20%, 2.17 ± 0.20%, 85.85 ± 1.98%, and 98.80 ± 2.24%, respectively. Clearly, the adsorption behavior fell into two distinct categories: non-Mg-modified biochars (SBC and K-SBC) and Mg-modified biochars (M-SBC and F-SBC). The Mg-modified biochars exhibited dramatically enhanced P adsorption, approximately 20 to 23 times greater than that of the raw biochar. Our findings support the mechanism proposed by Biswas et al., who likewise linked the enhanced adsorption capacity of Mg-modified biochars to higher magnesium content and greater pore volume [28]. Metal incorporation is known to promote physical adsorption, metal-ion complexation, and pore-filling mechanisms [29]. In contrast, K-SBC showed a 49.06 ± 5.10% reduction in adsorption capacity relative to SBC, likely due to enhanced surface reactivity that facilitated phosphate release without neutralizing the surface negative charge [30,31]. Equilibrium times also differed significantly. SBC and K-SBC reached saturation around 500 min, whereas M-SBC and F-SBC underwent rapid adsorption within 0–60 min, followed by a slower phase until equilibrium at 2160 min. Consequently, 2160 min was selected as the standard contact time for subsequent experiments.

Kinetic modeling (Table S1) indicated that the pseudo-first-order model better described P adsorption on SBC and K-SBC (R^2^ = 0.9860 and 0.9871), suggesting physisorption-dominated processes. In contrast, the pseudo-second-order model provided a superior fit for M-SBC and F-SBC (R^2^ = 0.9975 and 0.9881), indicating that chemisorption was the primary mechanism. This confirms that Mg^2+^-PO_4_^3−^ interactions play a crucial role in enhancing P adsorption on Mg-modified biochars, consistent with reports that high-P-adsorption biochars often follow pseudo-second-order kinetics [27].

Intra-particle diffusion analysis (Figure 2b and Table S2) revealed three-stage adsorption behavior as follows: (i) liquid film diffusion (t^0.5^ = 1~5.47 min), characterized by rapid surface site occupation; (ii) intraparticle diffusion (t^0.5^ = 5.47~30.98 min), where PO_4_^3−^ and HPO_4_^2−^ migrated into internal pores, with faster rates for Mg-modified biochars; and (iii) equilibrium (t^0.5^ = 30.98~46.47 min), where diffusion slowed due to site saturation. F-SBC showed the highest diffusion rate constant during the liquid film stage (K = 1.65 mg·g^−1^ min^−0.5^), which decreased in subsequent stages. The non-zero intercept (C = −3.44 to 13.82 ± 0.54 mg·g^−1^) in the Weber–Morris plot for F-SBC implies that both boundary layer and intraparticle diffusion contributed to rate control. However, the largest change in adsorption capacity (∆qt = 12.71 mg·g^−1^) underscores that intraparticle diffusion was the dominant rate-limiting step across all biochars, despite notable initial film diffusion effects [32,33].

#### 3.2.2. Adsorption Isotherms

The effects of initial P concentrations on the adsorption capacity onto different biochars are shown in Figure 2c. The adsorption capacity increased steadily in response to higher initial P concentrations. This trend is due to stronger interactions between the adsorbent and P at higher concentrations, coupled with an increased concentration gradient that enhances the driving force for adsorption, leading to more effective P removal from wastewater [34]. Although the adsorption capacity of SBC and K-SBC reached equilibrium at the initial P concentrations of 10 mg·L^−1^, that of M-SBC and F-SBC exhibited rapid increases until reaching adsorption equilibrium at the higher P concentrations of 50 mg·L^−1^. It is noticed that the adsorption capacity of P on M-SBC was less than that of F-SBC, which is possibly related to the lower Mg loading and small pore size, putting the limitation on the binding of Mg with absorbed P [35].

The isotherm adsorptions of various biochars are analyzed using Langmuir and Freundlich models to elucidate the adsorption mechanisms, and the corresponding fitted parameters are listed in Table S3. The Freundlich model exhibited higher correlation coefficients (R^2^) for SBC (0.9881), K-SBC (0.9859), while adsorption data of M-SBC and F-SBC fit better using the Langmuir model with R^2^ values of 0.9938 and 0.9915, respectively. This suggests that P adsorption occurs homogeneously as monolayers on the surfaces of M-SBC and F-SBC, while proceeding heterogeneously to form multimolecular layers on SBC and K-SBC surfaces. These findings align with Chen’s study, which demonstrated that metal ions enhance P adsorption onto biochars via complexation [36]. Furthermore, the suitability of the Langmuir model for describing the isotherm adsorption data supports the monolayer adsorption mechanism observed in metal-modified biochars. Additionally, the Freundlich model parameter 1/n < 2 confirms favorable adsorption conditions [37]. Notably, Guo et al. demonstrated that modified biochars with 1/n < 1 in Freundlich fittings possess heterogeneous binding sites, suggesting multi-mechanistic interactions [38].

### 3.3. Analysis of Influencing Factors

#### 3.3.1. Effect of Initial pH on the Adsorption Performance

**Figure 3:**
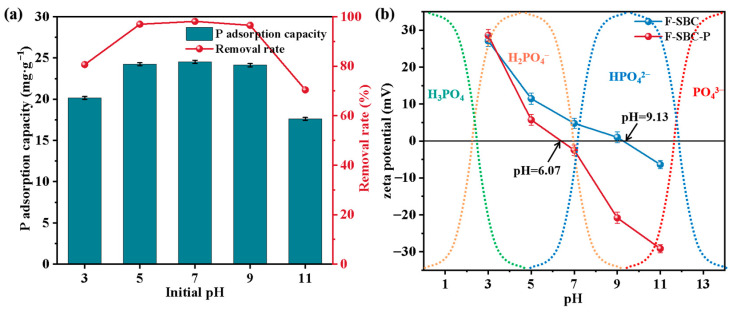
Effects of initial pH on phosphate adsorption (**a**) and pH_PZC_ (**b**) of F-SBC.

A further investigation into the influencing factors and applicability of F-SBC was conducted. As illustrated in Figure 3a, phosphate adsorption by F-SBC exhibited strong pH dependence, with adsorption capacities ranging from 17.61 ± 0.17 mg·g^−1^ to 24.70 ± 0.57 mg·g^−1^, corresponding to removal efficiencies of 70.42% to 98.80%. The maximum adsorption occurred under neutral pH conditions, while the efficiency declined to below 80% under highly acidic (pH < 3) or alkaline (pH > 11) conditions. This pH-dependent behavior can be attributed to phosphate speciation, surface charge variation, and competitive ion effects.

The stability and surface charge characteristics of MgO were strongly influenced by pH. Within the pH range of 5~9, the magnesium on the surface primarily existed as MgO and Mg(OH)_2_. The positive zeta potential indicated a positively charged surface, which resulted from the protonation of the surface hydroxyl groups (MgOH^+^ being the dominant species) in this pH range. The speciation of phosphate was also pH-dependent. Under mildly acidic to neutral conditions, the presence of protonated MgOH^+^ groups and H_2_PO_4_^−^ species favored electrostatic attraction, enhancing adsorption. In highly acidic media (pH < 3), however, adsorption capacity decreased due to two main factors: (1) the dominance of neutral H_3_PO_4_ (pKa = 2.12), which lacks electrostatic affinity, and (2) competitive inhibition from excess H^+^ ions. Under alkaline conditions (pH > 11), phosphate was present mainly as HPO_4_^2−^ (pKa = 12.67), and adsorption was hindered by both competition with OH^−^ ions and electrostatic repulsion caused by surface deprotonation (zeta potential: –6.19 mV at pH 11, Figure 3b) [39]. Nevertheless, at pH 9, F-SBC maintained 98% removal efficiency, likely due to the formation of stable inner-sphere complexes between Mg^2+^ and PO_4_^3−^.

F-SBC demonstrates broad pH adaptability, sustaining high phosphate removal (>95%) across a pH range of 5–9, with peak performance under neutral conditions. These properties highlight its potential as a robust adsorbent for P removal in constructed wetlands and other water treatment systems where pH fluctuations are common [4].

#### 3.3.2. Effect of Coexisting Anions on the Desorption Performance

**Figure 4:**
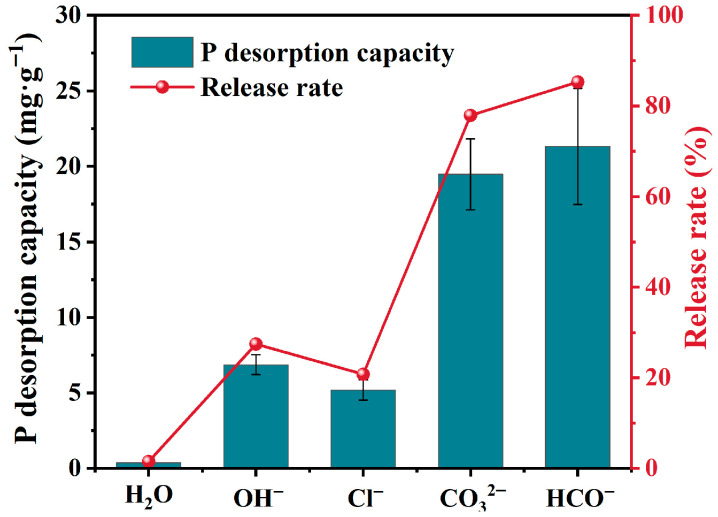
Effect of coexisting anions on the desorption performance of F-SBC.

The influence of coexisting anions (CO_3_^2−^, HCO_3_^−^, Cl^−^, OH^−^) on phosphate desorption from F-SBC was investigated through batch experiments (Figure 4). The P release rates in solutions containing OH^−^, Cl^−^, HCO_3_^−^, and CO_3_^2−^ were 31.56 ± 2.65%, 24.47 ± 2.63%, 85.29 ± 15.39%, and 78.90 ± 9.36%, respectively. The desorption efficiency followed the order HCO_3_^−^ > CO_3_^2−^ > OH^−^ > Cl^−^, revealing anion-specific competitive mechanisms [40]. HCO_3_^−^ and CO_3_^2−^ exhibited the strongest promotion effects, increasing phosphate release by 3.6–3.9 times compared to the control, consistent with findings from other studies on P release behavior in biochars [21,41,42,43]. This phenomenon may be attributed to the increase in solution pH resulting from the hydrolysis of CO_3_^2−^ and HCO_3_^−^. The elevated pH not only alters the surface properties of the adsorbent but also introduces competition from OH^−^, both of which inhibit the binding of PO_4_^3−^. OH^−^ induced release probably through direct site competition and surface charge reversal. Cl^−^ showed negligible impact, with only a 5.80~6.80% increase over control. This is consistent with low MgCl_2_ complexation stability and similar hydrated radii limiting competition. Therefore, the effect of coexisting ions should be considered alongside soil conditions when using biochar as a slow-release phosphorus fertilizer. The findings enable targeted biochar applications based on environmental chemistry.

#### 3.3.3. The Adsorption and Slow-Release Capacity Evaluation

**Figure 5:**
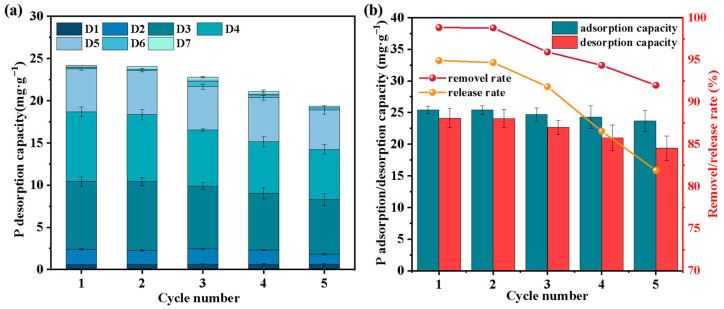
Effect of the number of cycles on the slow-release capacity (**a**), regenerative capacity (**b**) of F-SBC.

The slow-release behavior of F-SBC over five consecutive cycles is presented in Figure 5a. F-SBC exhibited notable phosphorus release performance, characterized by an initial rapid desorption phase between days 3 and 5, with an average release amount of 19.42 ± 1.35 mg·g^−1^. After day 6, the release amount declined to 0.32 ± 0.13 mg·g^−1^ and stabilized by day 7, indicating equilibrium. This release pattern is attributed to the rapid desorption of weakly surface-adsorbed phosphate followed by the slower diffusion of Mg^2+^-PO_4_^3−^ complexes from the inter-layer structure, as supported by previous spectroscopic analyses [44,45].

As illustrated in Figure 5b, the cumulative P release capacity decreased gradually from 24.07 ± 1.51 mg·g^−1^ to 18.40 ± 1.94 mg·g^−1^ over five cycles. Despite this decline in release performance, F-SBC maintained high structural and adsorptive stability. The adsorption capacity after multiple cycles remained at 23.12 ± 1.61 mg·g^−1^, preserving approximately 96% of the original value, while the P removal efficiency consistently exceeded 90%. The minimal loss in adsorption capability suggests that the active sites and structural framework of F-SBC are highly resilient under cyclic operation. The combination of sustained P release behavior and robust adsorption regeneration underscores the dual-function capability of F-SBC, thus positioning it as an ideal candidate for long-term phosphorus recovery and a controlled-release fertilizer.

### 3.4. Adsorption and Slow-Release Mechanism

**Figure 6:**
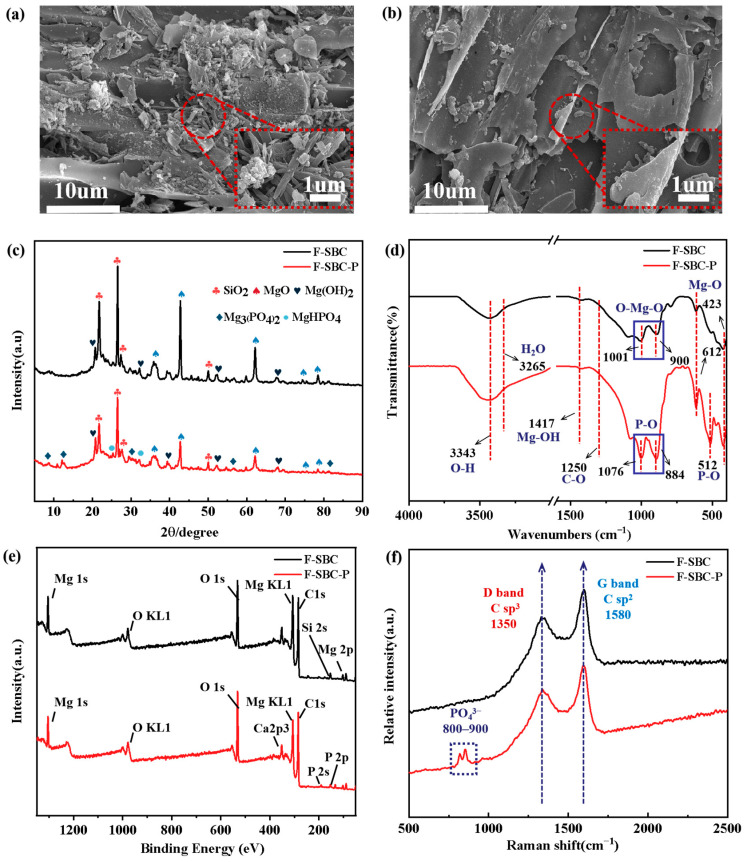
The changes in F-SBC before and after phosphate adsorption. (**a**) SEM images of F-SBC with magnification of 10 μm and 1 μm; (**b**) SEM images of F-SBC-P with magnification of 10 μm and 1 μm; (**c**) XRD, spectrum with Cu K_α_ radiation at the scan angle range of 5–90°, the scan speed at 5°/min; (**d**) FT-IR spectrum at wavelengths from 500 to 4000 cm^−1^ with the scan resolution at 4 cm^−1^; (**e**) XPS full survey spectra of excitation source in Al K_α_ rays (hv = 1486.6 eV) with a step of 0.1 eV; (**f**) Raman spectra at Raman shift from 500 to 2500 cm^−1^ with the scan resolution at 1 cm^−1^ of F-SBC and F-SBC-P.

Spectroscopic analyses (shown in Figure 6) revealed distinct structural and chemical transformations before and after phosphate absorption of F-SBC, providing direct evidence for each adsorption pathway.

XRD analysis confirmed the crystallographic evolution from MgO (characteristic peaks at 36.7°, 43.3°, and 62.5°) to crystalline phosphate species after P adsorption, including MgHPO_4_ and Mg_3_(PO_4_)_2_, evidenced by the emergence of new diffraction peaks at 18.6°, 32.8°, 37.9°, 50.8°, 58.6°, 62.0°, and 68.2° [46]. This transformation was accompanied by a 60% reduction in MgO peak intensity. Morphological transition from compact 4.2 nm MgO granules to larger rod-shaped phosphate crystals (6.0~9.5 nm) occupying interlayer spaces was observed. XPS quantification showed surface Mg decreased from 7.17 at.% (F-SBC) to 6.14 at.% (F-SBC-P), confirming Mg consumption. Surface-sensitive XPS (10 nm depth) detected higher surface P (3.12 at.%) than bulk EDS (0.69 at.%, 1 µm depth) (Table S4), indicating surface-dominated precipitation [47,48]. Quantitative XPS phase analysis (Figure S2) via Mg 1s showed the stoichiometric distribution of precipitated phases as Mg_3_(PO_4_)_2_ (47.17%), MgHPO_4_ (34.90%), and Mg(OH)_2_ (17.92%). This spatial distribution was further evidenced by comparative Mg:C ratios that XPS-derived surface measurements (10.70 at.%) aligned with theoretical calculations (9.11 at.%), while bulk EDS showed Mg content at 2.10 at.% (Tables S5 and S6). It is confirmed surface MgO/Mg(OH)_2_ preferentially initiates crystallization, extending sub-surface [49].

FT-IR spectroscopy revealed significant spectral changes upon phosphate adsorption. The Mg–O vibration at 423 cm^−1^ shifted to 612 cm^−1^, indicative of Mg–O–P bond formation, accompanied by the emergence of P–OH (512 cm^−1^) and P–O (884/1076 cm^−1^) vibrations and the disappearance of Mg–O bands in the 900–1000 cm^−1^ region. These changes confirm strong chemisorption and esterification-like reactions [40]. A redshift in O–H stretching from 3426 to 3413 cm^−1^ after P absorption, along with intensified absorption at 3265–3426 cm^−1^, suggested enhanced hydrate formation. XRD further corroborated the presence of crystalline hydrates such as Mg_3_(PO_4_)_2_·xH_2_O and MgHPO_4_·7H_2_O, supporting ligand exchange and inner-sphere complexation [50]. Surface stoichiometry derived from XPS (Mg:P ≈ 3:1) differed from bulk EDS (Mg:P ≈ 4:1), as shown in Table S7, reflecting nanoscale heterogeneity in Mg-P interactions [51]. High-resolution XPS (Figure S2) analysis revealed a decrease in C–O (284.0 eV) and C=O (285.0 eV) intensities post absorption, suggesting phosphate displacement of surface carbonates [52]. The O 1s spectra showed a reduction in Mg–O (530.9 eV) from 53% to 37% and an increase in Mg–OH/H_2_O (532.0 eV) from 47% to 62%, consistent with the appearance of a P 2p peak at 133.3 eV (attributed to C–O–P/O–P=O species). Chemical shifts in Mg 1s (−0.3 eV) and P 2p (−0.6 eV) binding energies affirmed the formation of stable inner-sphere Mg–O–P complexes after P absorption [53].

Zeta potential measurements indicated a positively charged F-SBC surface below pH 9.13, facilitating electrostatic attraction of phosphate anions (H_2_PO_4_^−^/HPO_4_^2−^) toward MgOH^+^ and Mg^2+^ sites [54]. Surface protonation underpinned this favorable electrostatic interaction, as evidenced by optimal phosphate removal at pH values below pHpzc. The detection of KOH (292.8 eV) shown in Figure S2 and residual K^+^ species identified by XPS peaks at 288.6 and 289.3 eV, suggested the formation of a weak bimetallic (Mg-K) adsorption layer. The weak bimetallic layer formed by K^+^ and Mg^2+^ contributed to additional electrostatic adsorption [55]. Structural characterization via XRD confirmed the presence of a graphitized SiO_2_ matrix (peaks at 20.4°, 26.6°, 50.2°). Raman spectroscopy showed an increase in the ID/IG ratio from 0.8252 to 0.8714 after adsorption, indicating an elevation in defect density within the carbon framework, consistent with pore filling and physical confinement of phosphate species.

## 4. Discussion

The enhanced performance of F-SBC compared to M-SBC can be attributed to the synergistic effect of KOH pre-activation followed by MgCl_2_ modification, which leads to improved textural properties and MgO dispersion. Although MgO deposition reduced the BET surface area of F-SBC compared to K-SBC, KOH pretreatment significantly expanded the pore structure, resulting in a larger average pore diameter and higher pore volume than SBC. This facilitated superior MgO dispersion, as confirmed by EDS, which showed higher Mg content in F-SBC. XRD revealed sharper MgO/Mg(OH)_2_ peaks in F-SBC, indicating enhanced crystallinity and surface reactivity. FT-IR further confirmed intensified Mg-O and Mg-OH vibrations, reflecting a higher density of active sites for phosphate chemisorption.

In contrast, K-SBC exhibited lower phosphate adsorption capacity than SBC, likely due to structural instability. The corn straw biochar, rich in cellulose, hemicellulose, and lignin, undergoes hydrolysis during KOH treatment, leading to structural variation. FT-IR results support this, showing cleavage of ester linkages, with the band at 1819 cm^−1^ converting to 1695 cm^−1^. It is hypothesized that this alkaline induced instability may cause intrinsic phosphorus release during adsorption experiments. Furthermore, the efficacy of phosphorus adsorption by alkali-modified biochar is governed by a complex interplay of factors, including the properties of the carbonaceous base material, KOH concentration, activation temperature, and duration. The complex phenomenon that merits further investigation.

**Figure 7:**
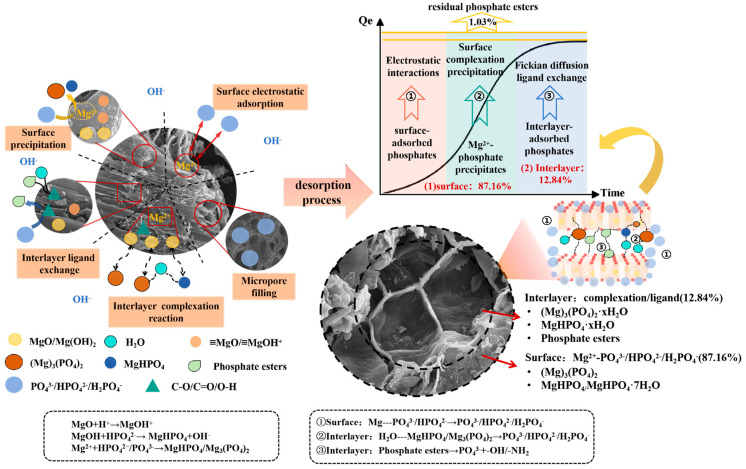
The mechanisms of phosphate adsorption and slow release on F-SBC.

The phosphorus adsorption and slow release behavior of F-SBC are governed by its hierarchical architecture, which features a graphitized SiO_2_ matrix intercalated with Mg based crystallites. The process of absorption is driven primarily by chemical precipitation and chemisorption, supplemented synergistically by other mechanisms like inner-sphere complexation, electrostatic interaction, and physical confinement. The release process occurs in two stages: a surface dominated stage involving dissolution of MgHPO_4_ and related hydrates at pH above 6.19, followed by a gradual decomposition stage of subsurface Mg_3_(PO_4_)_2_·xH_2_O and stable phosphate esters, enabling sustained phosphorus release [56,57,58,59]. BET analyses indicate that Mg-P precipitates progressively occupy pore spaces, moderating intraparticle diffusion. XPS identification of C–O–P species further suggests an equilibrium controlled interlayer release pathway. This multimechanistic framework explains both the high adsorption capacity and sustained release profile of F-SBC, providing a foundation for optimizing its application in phosphorus recovery and slow release fertilization. The mechanisms of P adsorption and slow release on F-SBC are shown in Figure 7.

F-SBC exhibits high P-release capacity (24.07 mg·g^−1^), surpassing Mg/Al-biochars (3.87 mg·g^−1^), Mg-sludge biochars (2.44 mg·g^−1^), and Zn/Mg-biochar (19.13 mg·g^−1^) [56,57,58]. Analogous modified biochars enhance root development from 5.58 cm to 7.32 cm, increase dry biomass by 10–19%, and boost plant P utilization efficiency by 17% [15,57,59]. The high phosphorus availability and significant biomass enhancement conferred by F-SBC-P establish it as a promising candidate for a high-performance fertilizer. These compelling results validate the need for further implementation studies to translate these findings into practical agricultural applications.

## 5. Conclusions

This study demonstrates the successful development of KOH/MgCl_2_-modified F-SBC for P recovery and slow-release applications. Through KOH activation, F-SBC achieved a 23-fold enhancement in P adsorption capacity compared to raw biochar, attributed to expanded pore structures, improved MgO loading, and synergistic mechanisms involving chemical precipitation, surface complexation, electrostatic interactions, and physical confinement. Remarkably, F-SBC maintained the high P removal efficiency across a pH range of 5~9, with optimal neutral pH performance due to stable MgO and favorable H_2_PO_4_^−^ electrostatic attraction. The material exhibited excellent practical potential, demonstrating phased P release and cyclic stability. This work not only advances biochar engineering but also provides a feasible solution for sustainable phosphorus management. Future studies should prioritize field trials and lifecycle assessments to validate applicability.

## Figures and Tables

**Table 1:** Parameters from BET analysis and the elemental composition from EDS.

Sample	BET (m^2^·g^−1^)	Pore Volume (cm^3^·g^−1^)	Pore Diameter (nm)	At.%					
				C	O	Mg	Si	K	Ca
SBC	177.3884	0.1031	2.3244	87.4	10.4	0	1.7	0	0.5
K-SBC	197.0262	0.1118	2.2697	81.8	12.4	0	2.6	2.3	0.8
M-SBC	164.5370	0.0951	2.3119	82.0	9.8	6.1	1.8	0	0.2
F-SBC	137.6233	0.1051	3.0545	81.1	10.5	6.9	1.0	0.4	0.1

## Data Availability

The original contributions presented in this study are included in the article/Supplementary Materials. Further inquiries can be directed to the corresponding author.

## Supplementary Materials

The following supporting information can be downloaded at: https://www.mdpi.com/article/10.3390/ma18225214/s1, Figure S1: Nitrogen adsorption–desorption isotherms and BJH pore size distribution degassing for 4 h in a vacuum at 300 °C of raw material, SBC (a); modified materials, K-SBC(b), M-SBC(c), and F-SBC (d); Figure S2: XPS spectrum of F-SBC and F-SBC-P. (a) C 1s, (b) Mg 1s, (c) O 1s, (d) P 2p, and (e) K 2p; Table S1: Fitting parameters for adsorption kinetics of different materials; Table S2: Fitting parameters for intra-particle diffusion kinetics of different materials; Table S3: Fitting parameters for adsorption isotherms of different materials; Table S4: Content analysis of XPS; Table S5: Content analysis of EDS; Table S6: Mg and P mol% of EDS analysis, XPS analysis, and theoretical calculation; Table S7: Phase analysis of XRD.
