# Supplementary material for: Unraveling the Synergistic Mechanisms of Phosphorus Adsorption and Slow-Release on Low-Mg-Loaded Biochar Enabled by KOH Activation
Source: Materials (Basel). 2025 Nov 18;18(22):5214. doi: 10.3390/ma18225214 (PMC12654471; doi:10.3390/ma18225214)
Supplement: Supplementary file 1 [file materials-18-05214-s001.zip › materials-3941780-supplementary.pdf]

Table S1. Fitting parameters for adsorption kinetics of different materials.

Sample	Adsorption kinetic equation					
	Pseudo-first-order			Pseudo-second-order		
	Q_e/mg·g^{-1}	K_1/min^{-1}	R^2	Q_e/mg·g^{-1}	K_2/g·mg^{-1}min^{-1}	R^2
SBC	1.49	0.0014	0.9860	1.53	0.0347	0.8092
K-SBC	0.58	0.0019	0.9871	0.60	0.2035	0.9070
M-SBC	21.94	0.0018	0.9192	21.48	0.0098	0.9975
F-SBC	24.94	0.0021	0.9237	24.75	0.0019	0.9981

Table S2. Fitting parameters for intra-particle diffusion kinetic of different materials.

Sample	Stage	K_{id}/g·mg^{-1}min$^{-0.5}$	R^2	C/mg·g^{-1}
	I	0.17813	1	-0.15094
SBC	II	0.02343 ± 0.00395	0.97548	0.92822 ± 0.0709
	III	0.00133 ± 0.00044	0.9999	1.44048 ± 0.01886
	I	0.00741	1	0.17509
K-SBC	II	0.01289 ± 0.00132	0.95039	0.15996 ± 0.02663
	III	-0.00046 ± 0.00021	0.94328	0.52795 ± 0.00921
	I	0.90091	1	-2.09533
M-SBC	II	0.57565 ± 0.01041	0.99837	-0.7462 ± 0.20677
	III	0.24349 ± 0.00322	0.99983	10.7149 ± 0.13636
	I	1.64774	1	-3.43741
F-SBC	II	0.56973 ± 0.02273	0.99211	3.67807 ±0.39389
	III	0.21903 ± 0.01275	0.99662	13.81925 ± 0.53691

Table S3. Fitting parameters for adsorption isotherms of different materials.

Sample	Langmuir model			Freundlich model		
	Q_m/mg·g^{-1}	K_L/L·mg^{-1}	R^2	K_F/(mg·g^{-1})·(mg·L^{-1})$^{-n}$	1/n	R^2
SBC	1.51	0.2215	0.9482	52.0979	0.3929	0.9881
K-SBC	0.69	0.0454	0.9037	9.2504	0.6079	0.9859
M-SBC	22.42	0.2584	0.9938	2.1299	0.4787	0.8626
F-SBC	25.13	0.1228	0.9915	5.3142	0.3704	0.8958

Table S4. Content analysis of XPS.

Sample	Element	Structure	Proportion(%)	Atom%	Variation of At% Between F-SBC and F-SBC-P
		C-C	53.76		
	C	C-O	32.80	67.01	-
		C=O	13.44		
	O	O-H	38.65	24.9	-
F-SBC		Mg-O	61.35		
	P	-	-	0.76	-
	K	KCl	-	0.15	-
	Mg	Mg-O	52.08	7.17	-
		Mg-OH	47.92		
		C-C	49.50		
	C	C-O	33.17	65.58	-1.43
		C=O	13.86		
		C=O-C	3.47		
	O	O-H	49.24	25.87	0.97
		Mg-O	50.76		
F-SBC-P		$Mg_3(PO_4)_2$	55.25		
	P	$MgHPO_4$	43.72	2.05	1.29
		C-O-P	1.03		
	K	KOH	-	0.35	0.2
		$Mg_3(PO_4)_2$	47.17		
	Mg	$MgHPO_4$	34.90	6.14	-1.03
		$Mg(OH)_2$	17.92		

Table S5. Content analysis of EDS.

Sample	Element	Atom%	Variation of At% Between F-SBC and F-SBC-P
	C	86.91	-
	O	9.17	-
F-SBC	P	0.31	-
	K	0.10	-
	Mg	2.10	-
	C	72.68	-14.23
	O	23.17	14
F-SBC-P	P	0.50	0.19
	K	0	-0.1
	Mg	1.80	-0.3

Table S6. Mg and P mol% of EDS analysis, XPS analysis and theoretical calculation.

Element	Method	Dosage	Relative molecular mass	Atom%	Ratio to C(%)
C of F-SBC	Theoretical content	1g	12.01	-	-
	Surface(XPS)	-	-	67.01	-
	Subsurface(EDS)			86.91	-
Mg of F-SBC	Theoretical content	25mL	24.305	-	9.11
	Surface(XPS)	-	-	7.17	10.70
	Subsurface(EDS)	-	-	2.10	2.41
P of F-SBC-P	Theoretical content	50mL	30.97	-	2.50
	Surface(XPS)	-	-	2.05	3.12
	Subsurface(EDS)	-	-	0.50	0.69

Table S7. Phase analysis of XRD.

Sample	Phase	Wight%	Content variation%	Grain size(nm)
F-SBC	MgO	5.40		4.15
	SiO_2	84.40		4.68
	$Mg(OH)_2$	3.00		5.58
	P_2O_5	1.10		
	$C_{15}H_{24}O_2$			
	$C_{14}H_8O_4$	6.00		
	$C_7H_6O_3$			
F-SBC-P	MgO	4.10	-1.30	4.15
	SiO_2	80.10	-4.30	4.68
	$Mg(OH)_2$	2.60	-0.40	5.58
	$Mg_3(PO_4)_2$	3.50	3.50	7.00
	$MgHPO_4 \cdot 7H_2O$	2.90	2.90	9.50
	$Mg_3(PO_4)_2 \cdot xH_2O$	0.90	0.90	9.40
	Mg_2PO_4OH	0.10	0.10	6.00
	$C_{15}H_{24}O_2$			
	$C_{14}H_8O_4$	5.00	-1.00	
	$C_7H_6O_3$			

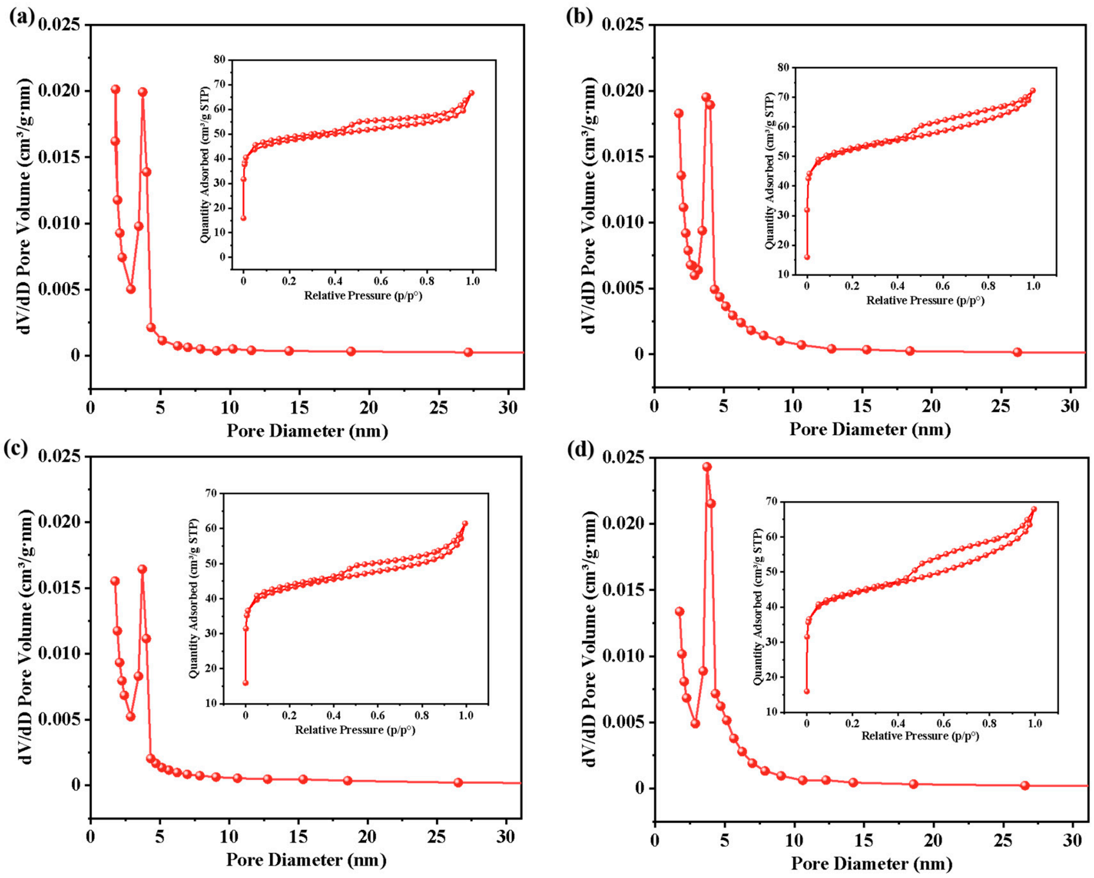

Figure S1. Nitrogen adsorption-desorption isotherms and BJH pore size distribution degassing for 4 h in a vacuum at 300 °C of raw material, SBC (a); modified materials, K-SBC(b), M-SBC(c), and F-SBC (d).

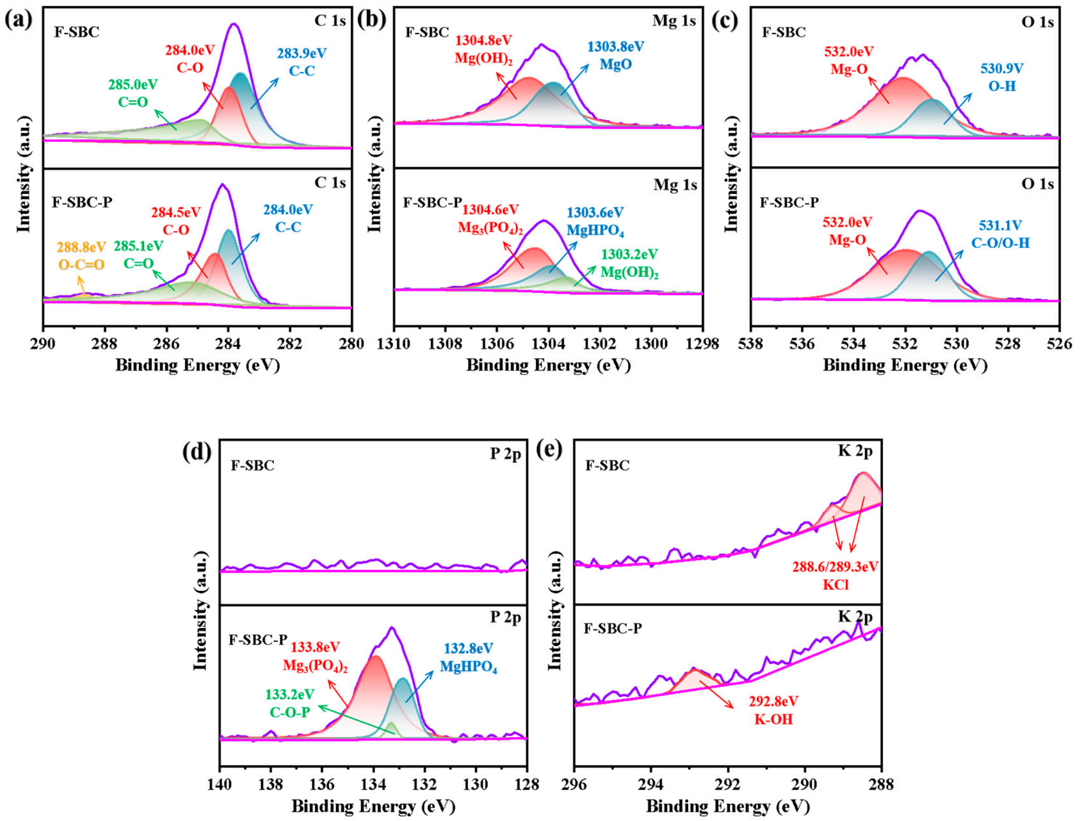

Figure S2. XPS spectrum of F-SBC and F-SBC-P. (a) C 1s, (b) Mg 1s, (c) O 1s, (d) P 2p and (e) K 2p.
